# Patients’ experiences of penicillin allergy evaluation: a qualitative study

**DOI:** 10.1093/jacamr/dlaf261

**Published:** 2026-02-04

**Authors:** Ágnes Csuth, Linda Gustafsson Wännlund, Maria C Jenmalm, Lene Heise Garvey, Charlotte Angelhoff

**Affiliations:** Division of Inflammation and Infection, Department of Biomedical and Clinical Sciences, Linköping University, Linköping, Sweden; Clinical Department of Allergy Center in Linköping, Region Östergötland, Linköping, Sweden; Clinical Department of Allergy Center in Linköping, Region Östergötland, Linköping, Sweden; Department of Health, Medicine, and Caring Sciences, Linköping University, Linköping, Sweden; Division of Inflammation and Infection, Department of Biomedical and Clinical Sciences, Linköping University, Linköping, Sweden; Clinical Department of Allergy Center in Linköping, Region Östergötland, Linköping, Sweden; Allergy Clinic, Department of Dermatology and Allergy, Copenhagen University Hospital-Herlev and Gentofte, Copenhagen, Denmark; Department of Clinical Medicine, University of Copenhagen, Copenhagen, Denmark; Clinical Department of Allergy Center in Linköping, Region Östergötland, Linköping, Sweden; Department of Health, Medicine, and Caring Sciences, Linköping University, Linköping, Sweden

## Abstract

**Background and objectives:**

Penicillin allergy is often overdiagnosed, with a 10% reported prevalence in affluent countries. Incorrect labels lead to broad-spectrum antibiotic use, longer hospital stays and MDR infections. Understanding patients’ perspectives is crucial to enhance de-labelling and ensuring penicillin use when indicated. To describe patients’ experiences of being labelled as allergic to penicillin and their willingness to take penicillin after a negative challenge.

**Patients and methods:**

Fifteen patients referred for allergy investigation were included and participated in semi-structured interviews, regardless of allergy risk or subsequent evaluation results. The data were analysed with qualitative content analysis using the Graneheim and Lundman approach.

**Results:**

Three main categories were identified: ‘Frustration over insufficient documentation and communication’, ‘Factors that determine whether participants want to undergo a drug challenge’ and ‘What happens after drug challenge? Willingness to accept penicillin after the allergy work-up’. Poor documentation led to insecurity. Trust in healthcare professionals and awareness of the negative consequences of allergy labels contributed to participants’ acceptance of drug challenges. The participants were willing to take penicillin after a negative challenge, although some preferred the first dose of subsequent treatments to be administered close to advanced healthcare infrastructure.

**Conclusions:**

Improved and comprehensive guidelines for the management of suspected penicillin allergy are necessary to enhance understanding of penicillin allergy and ensure that patients are promptly evaluated after a suspected allergic reaction with referral to an allergist if indicated.

## Introduction

Penicillin allergy (PA) is frequently overdiagnosed, with a reported prevalence up to 10% in affluent countries. Prior studies indicate that approximately 9 out of 10 penicillin allergy labels (PALs) can be dismissed following systematic evaluations, including drug challenge tests.^1–3^ Carrying a PAL can lead to numerous adverse consequences, such as overuse of broad-spectrum antibiotics that might be less effective, cause more side effects, extend hospital stays, and elevate the risk of infections caused by MDR bacteria.^1,4–6^ Several factors contribute to the recording of PALs beyond genuine hypersensitivity reactions, including infections and mild non-allergic side effects, such as nausea, diarrhoea and thrush.^3,7,8^ Many labels refer to vague past reactions, with some lacking any details of the initial event. Although the decline of specific IgE to penicillin can facilitate de-labelling, this may not always indicate tolerance, and resensitization has been reported.^9,10^

Sweden, along with other Scandinavian countries, is recognized for its stringent antibiotic policy and the use of narrow-spectrum penicillins as first-line antibiotics for several community-acquired infections. Sweden ranks among the lowest globally in both antibiotic consumption and antibiotic resistance rates.^11–14^ This achievement is largely attributable to the Strama Network (Collaboration against Antibiotic Resistance in Sweden), a nationwide initiative aimed at combating antibiotic resistance within healthcare. The Strama Network plays a crucial role in monitoring antibiotic usage, implementing clinical guidelines, and educating physicians about these guidelines and the importance of removing incorrect PALs.^15^

Despite the existence of international and national guidelines for evaluating suspected penicillin allergies and physician awareness of the adverse consequences associated with incorrect allergy labelling, a significant proportion of patients do not undergo PA evaluation. Contributing factors include unclear information about the initial reactions, varying interpretations of immunological reactions, controversial recommendations in the guidelines and the shortage of allergy specialists. Studies from outside Sweden dealing with long term follow-up of de-labelled patients indicate that a significant number of patients decline treatment with penicillin despite an uneventful drug challenge test.^16,17^ Therefore, addressing the patients’ perspective and concerns is essential to ensure that de-labelling is successful, and in convincing the patients to take the relevant medication if it is indicated.

To date, limited information exists regarding patients’ perspectives when they are suspected of having a PA. An interview study conducted in the UK revealed that the individuals most willing to undergo PA diagnostic testing were those who had experienced negative consequences from being labelled as allergic to penicillin, such as being denied first-line treatment and having limited antibiotic choice.^18^ Additionally, a recent prospective longitudinal cohort study, conducted on participants registered in the US Drug Allergy Registry, indicated that the vast majority (83%) of patients expressed concerns about their drug allergies. Factors associated with greater patient concerns included increased age, having a higher number of reported drug reactions, experiencing itching in the mouth or palate, and requiring antibiotics more frequently.^19^

According to a Danish study, the most common reason for hesitation to take penicillin was fear of an allergic reaction.^1^ Only 19% responded that they would take penicillin outside of the allergy clinic before a drug challenge test; however, all patients affirmed they would take penicillin after an uneventful prolonged drug challenge test.^1^ Given that allergy assessments can be time-consuming and resource intensive, it is critical to consider patients’ perspectives and concerns in order to enhance the efficiency of de-labelling and to ensure that patients are willing to accept the relevant medication when indicated. The aim of this study was to describe patients’ experiences of being labelled as allergic to penicillin and their willingness to take penicillin after a negative challenge test.

## Patients and methods

### Ethics

This study was approved by the Swedish Ethical Review Authority (Stockholm, Department 4 Medicine, 2024/01835-01), and complied with the Declaration of Helsinki.^20^

### Design

This was a qualitative, descriptive study with an inductive and exploratory approach. The study is part of a larger study exploring de-labelling of penicillin allergies.

### Participants and procedure

Inclusion criteria were Swedish-speaking patients over 18 years old, either referred by primary care physicians or various specialist clinics to the Allergy Center for evaluation of PA, or patients being assessed at the Allergy Center for other allergies but carrying a PAL or reporting a suspected PA. Exclusion criteria were prior penicillin allergy evaluation by another allergist, severe delayed skin reaction or organ failure from penicillin, and pregnancy or breastfeeding.

Twenty eligible patients were identified and underwent risk stratification by an allergy specialist (A.C.) according to updated Danish guidelines^1^ (see Table S1 and Supplementary material, available as Supplementary data at *JAC-AMR* Online). Three patients declined participation at this stage. Interviews were scheduled with 17 individuals after obtaining informed consent. Two patients who agreed to participate did not answer the phone calls at the scheduled time, despite a couple of attempts to reschedule, and were therefore excluded from the study. Ultimately, 15 interviews were conducted.

### Data collection

Fifteen patients (10 female and 5 male) aged 22–71 years (median age 52 years) participated in the study. At the time of the interviews, two patients had undergone drug challenges, including prolonged challenges, which yielded negative results. Two participants with serious immediate-type reactions had been informed about having positive specific IgE assays towards penicillin at the time they were interviewed. Since a positive IgE test combined with a compatible clinical history is strongly indicative of an IgE-mediated penicillin allergy, these patients were classified as penicillin-allergic without undergoing a drug challenge (Table 1).^21,22^

**Table 1.** Description of participants

	*n*
Participants already evaluated at the time of interviews	4
Negative drug challenge test	2
Penicillin allergy confirmed without challenge test	2
Severe systemic reaction as index reaction	2
Non-allergic side effects	1
Exclusively skin symptoms (maculopapular exanthema, urticaria, angioedema)	12
Distant reaction as a child	5
Reaction both in childhood and adulthood	1
Reaction in adulthood	9
Referred from other clinic due to suspected allergy to penicillin	10
Referred due to concomitant allergic disease and had a penicillin allergy label	5
Symptoms consistent with IgE-mediated reactions of any severity	10

Semi-structured interviews were conducted between August and October 2024 by C.A., skilled in qualitative research methodologies and independent of the patients’ clinical evaluation or further assessment. The interviews followed a semi-structured guide, comprising six questions, devised by A.C. and C.A. (Table 2).

**Table 2.** Interview guide

1. Tell me about when you had an allergic reaction to penicillin What happened? When was this?
2. How have you processed that event afterwards?
3. Has your penicillin hypersensitivity had an impact on your everyday life? How? Example, tell me more
4. How have you experienced the evaluation that has been performed? What has been done? How was the interaction with the healthcare staff?
5. How do you feel about performing a penicillin challenge at the Allergy Center? What do you think about it? Preparations?
6. If an evaluation showed that you are not allergic to penicillin, would you accept to have penicillin prescribed if the need arose? Why/why not?

Most participants (*n* = 12) preferred telephone interviews to avoid travelling to the outpatient clinic; however, three participants opted for face-to-face interviews, which were conducted at the Allergy Center. All interviews were audio recorded and transcribed verbatim. The interviews had an average duration of 16 min.

### Data analysis

Content analysis of the data was conducted according to the Graneheim and Lundman approach.^23^ First, two interviews were independently coded by three authors (A.C., L.G.W. and C.A.), and their codes were compared to validate coding consistency. Subsequently, A.C. and L.G.W. coded the remaining interviews (Table 3). After 10 interviews, an initial analysis was conducted (A.C., L.G.W., C.A.), revealing that the codes, which were identified and developed from the interview material, were fully understood. During analysis we observed that no new aspects, dimensions or nuances emerged after the 10th interview. The remaining five interviews confirmed this saturation. As the sample was specific, the interviews provided valuable and reasonably detailed material, and the dataset was coherent, we achieved trustworthiness.^23,24^ During data analysis, an inductive coding approach was used.

**Table 3.** Example of content analysis process

Condensation of answers	Codes	Subcategories	Descriptive category
‘They (the caregivers) do not take it seriously, as a patient I have to remind them of my suspected allergy.’	The patients’ responsibility to inform the caregivers about the suspected penicillin allergy	Insecurity in patients due to the insufficient documentation of the suspected allergy	Frustration over insufficient documentation and communication
‘Yes, I wanted to perform it (drug challenge test). Penicillin is the first-line antibiotic treatment and why should I expose myself or my gut flora for broad-spectrum antibiotics unnecessarily?’	Avoiding broad-spectrum antibiotics is a motivating factor to accept drug challenge test	The importance of the negative consequences associated with the penicillin allergy labels and getting a conclusion	Factors that determine whether participants want to undergo a drug challenge
‘I feel very safe because I know that it will be documented in my chart, so the caregivers can see it as a warning.’	Correct documentation after allergy work-up is important	Labelling or de-labelling? The correct documentation is crucial regardless of the outcome	What happens after drug challenge? Willingness to accept penicillin after the allergy work-up

## Results

**Figure 1.**
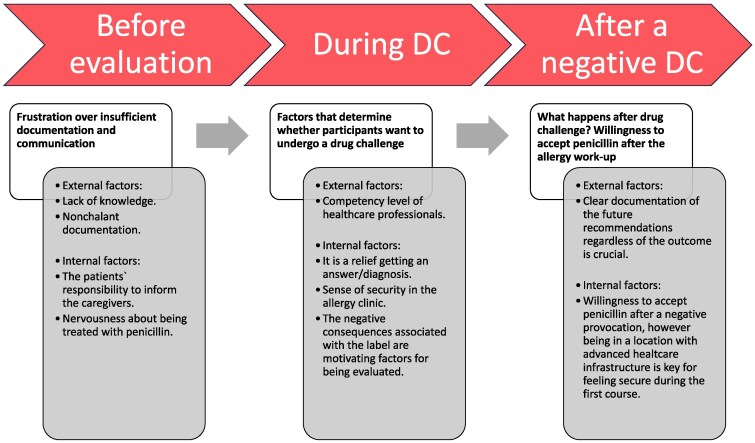
Timeline of participants’ experiences during the penicillin allergy work-up. Key phases in the patient journey, highlighting external and internal factors that influenced participants’ experiences. Categories are positioned along a timeline to reflect how these factors evolved before, during and after the drug challenge. DC, drug challenge.

Three main categories were identified: ‘Frustration over insufficient documentation and communication’, ‘Factors that determine whether participants want to undergo a drug challenge’ and ‘What happens after drug challenge? Willingness to accept penicillin after the allergy work-up’, with the subcategories as external and internal factors (Figure 1).

### Frustration over insufficient documentation and communication

This category described how the caregivers treated and evaluated patients with a suspected PA and the participants’ experiences regarding the documentation of their suspected allergic reactions to penicillin. The documentation of the PA was lacking or incomplete in several cases, and referral for allergy work-up was not routinely offered. The patients felt that it was their responsibility to inform the caregivers about their suspected allergy.

#### Poor knowledge about penicillin allergy and insufficient documentation by the healthcare professionals

The suspected PA was not properly documented, and penicillin was prescribed despite reported reactions to penicillin in several cases. Respondents with late-onset symptoms explained that reactions that occurred days after the first dose were not interpreted as possible allergic manifestations and were deemed as non-allergic side effects or manifestations of another disease in numerous cases. A female patient with recurrent reactions that developed several days after the therapy start experienced increasing severity grade every time she was treated with penicillin, and after the third occasion she got serious angioedema and breathing difficulties. She had visited her primary care centre after the first and the second reactions, and had been evaluated for suspected allergy to airborne allergens; contact allergy had also been suspected. These interpretations led to frustration and doubt in the caregivers generally. A male patient with a PAL due to a severe systemic reaction expressed discontent when he was interviewed, explaining that his previous reaction was repeatedly questioned by healthcare personnel and penicillin was offered several times, despite the label.

It could have killed me. Honestly, I just wonder how many people they kill every year, because they simply do not know, because there are hypersensitivities they just cannot handle, because at the emergency unit they cannot handle it. (pc 12)

Most patients were not informed about the negative consequences associated with a PAL and were referred to an allergist several years or decades after the initial reaction or when the suspected PA led to difficulties, e.g. in choice of antibiotics.

In some cases, the patients themselves requested referrals for an allergy work-up, and a referral was not routinely offered by the healthcare professionals. In a significant number of patients, broad-spectrum antibiotics were prescribed without discussing the patients’ history.

#### Insecurity in patients due to insufficient documentation of the suspected allergy

Given that the documentation of the suspected PA was not correct or lacking, several patients pointed out that they felt it was their responsibility to inform the caregivers about the previous reactions. This led to the sense of insecurity in the participants, who found it difficult to double-check the prescriptions. In some cases, penicillin was prescribed even though the patients had earlier discussed with the prescribing doctor about the suspected PA. That prompted the patients to scrutinize every time antibiotics were dispensed and check the ingredients carefully.

I can feel a bit uncomfortable when I notice that the doctors approach me … that they are a little nervous, because they do not know what to give me instead. They just do not know. Yeah, so quite often I check what I have been given to make sure it is not just another sort of penicillin, like. I mean, I have actually been given penicillin even though I was obviously labelled as being allergic. (pc 1)

One participant explained frustration about situations when it was difficult to get prescriptions for antibiotics, because some caregivers did not want to prescribe alternative broad-spectrum antibiotics, and it led to delayed treatment and prolonged symptom duration.

### Factors that determine whether participants want to undergo a drug challenge

This category described the crucial role of healthcare professionals’ competency level regarding the drug challenge tests and which factors motivated the participants to accept a drug challenge.

#### Healthcare professionals’ role in drug challenge and patient safety

The healthcare professionals’ competency had a great impact on the patients’ attitude towards the drug allergy work-up, including drug challenge tests. Several patients described that they had trust in the healthcare professionals who performed the evaluation, and this trust was crucial in accepting to go further with a drug challenge test, if necessary. A few patients who had chronic allergic diseases and had been patients in the allergy clinic for a longer period explained that it was important that their allergist was familiar with their whole history, and it increased their trust in the healthcare personnel of the clinic. These factors contributed to the sense of feeling safe during the whole drug allergy evaluation.

I was not too nervous about something happening to me. I knew that if something had happened, the competence was there to help me. (pc 4)

#### Importance of the negative consequences associated with the PALs and getting a conclusion

Most of the participants felt safe and were not particularly anxious about the upcoming drug challenge test. Those who had already undergone it felt safe during the whole evaluation and expressed thankfulness. Some patients mentioned that it was nice to get a conclusion after several years of insecurity. The respondents described that the harmful consequences of carrying a PAL, such as overuse of broad-spectrum antibiotics leading to MDR bacteria, and difficulties in the choice of antibiotics, were taken into consideration when they agreed to take a drug challenge test. The participants pointed out that they wanted to avoid insecurities in the future caused by a suspected PA and that they wanted to perform the drug challenge electively in a safe setting.

So, I was really happy when I was able to test it and get a clear answer in black and white, whatever the results. So, yes, it was good. (pc 1)

### What happens after drug challenge? Willingness to accept penicillin after the allergy work-up

This category focused on the importance of the correct documentation regardless of the outcome of the allergy work-up. It also highlighted that most patients were willing to take penicillin after a negative drug challenge; however, some of them emphasized that the proximity of healthcare infrastructure was essential for feeling safe the first time penicillin was prescribed after the label had been removed.

#### Labelling or de-labelling? The correct documentation is crucial regardless of the outcome

Most patients underlined that clear documentation regardless of the results was more important than the outcome itself. Especially, those who had tried being treated with or got prescriptions for penicillin despite carrying a PAL emphasized that the future documentation after the evaluation must be correct and easy to understand.

The two patients who had already been informed about having positive specific IgE assays towards penicillin also underlined that correct documentation of the PA was the most important factor for them to feel safe if antibiotics were prescribed in the future. A male patient with confirmed PA who had suffered a systemic allergic reaction previously pointed out that it was a relief to get the label so he could avoid being ‘questioned’.

Some participants felt nervous because of the poor documentation. They expressed that it was essential that healthcare professionals would scrutinize allergy labels and the medical notes regarding the performed drug allergy evaluations before prescribing or administering medications in future.

The allergist told me that it would be documented in my records. Doctors do not always listen when you say something, so I do not want to get something that makes my heart stop beating. I heard that it could affect the internal organs … and it can go very fast. (pc 3)

#### Motivation and willingness to accept penicillin after the drug challenge

Participants who were still waiting for an evaluation and those who had already undergone challenges stated that they would accept penicillin after a negative challenge. Most respondents did not express doubt regarding being treated with penicillin in the future. One participant, with a moderate skin reaction occurring at the end of the penicillin course, was willing to undergo a penicillin challenge but hesitant about long-term treatment.

Those participants who felt unsafe during antibiotic treatment, due to not being labelled as PA after their initial reaction and still awaiting evaluation, reported frustration with poor documentation of allergy labels but were willing to undergo a supervised allergy evaluation, including drug challenge. They also stated that they would take penicillin after a negative challenge, while remaining more observant and cautious. Others pointed out that they would only feel safe in taking penicillin again if the first doses or the first course could be taken in a location with advanced healthcare infrastructure due to anxiety related to previous negative experiences.

Of course, I would be more observant if I took penicillin in the future. I would probably not go to the countryside, I would rather be close to a city … I would like to be near health care the next time I need to take a full course of penicillin. (pc 13)

## Discussion

This study aimed to explore patients’ experiences of being evaluated for a suspected PA and their willingness to take penicillin after a negative drug challenge. Our findings indicate that the patients felt frustration over the lack of correct understanding and documentation of PALs by healthcare professionals. They also revealed that patients’ awareness of the potential harms of inaccurate labels affects their willingness to undergo evaluation and accept de-labelling. In addition, patients expressed willingness to take penicillin after a negative challenge and preferred to receive support from well-equipped healthcare services during their first use post-de-labelling.

Our findings align with previous studies that indicate poor understanding of penicillin allergies by non-allergist physicians and that clinicians had limited experience of the referral procedure. Many of them lacked knowledge about the allergy work-up.^18,25,26^

To address this, targeted educational initiatives and standardized referral procedures should be implemented to ensure accurate assessment, appropriate de-labelling and safer antibiotic prescribing. Non-allergists should be made more aware of the significance of labelling and familiarize themselves with updated risk evaluation guidelines. It is important to emphasize that non-allergists require structured support to safely and effectively undertake diagnostic challenges aimed at de-labelling using direct oral challenge.^27^ Without such guidance, opportunities to reduce inappropriate allergy labelling may be missed, particularly in clinical settings where access to allergy specialists is limited. Providing clear protocols, training and collaborative frameworks can enable non-allergists to contribute meaningfully to de-labelling efforts, thereby improving patient care and reducing unnecessary restrictions.

In line with other studies, many of our participants were unfamiliar with the negative consequences of having a PAL and were referred for allergy evaluation decades after the index reaction.^18,28^ Broad-spectrum antibiotics were often prescribed without discussing the suspected reaction and potential disadvantages with patients. Moreover, referrals were frequently made only when it was difficult to prescribe the most effective antibiotics or when the patients requested referral to an allergy specialist. Many participants were also unaware that allergy evaluation was an option until they were referred to the allergy clinic. Therefore, to avoid overusing broad-spectrum antibiotics, timely referral for suspected allergic reactions should be encouraged. This would allow for early assessment and de-labelling when appropriate.

Furthermore, informants also described a lack of awareness that the diagnosis of PA could be incorrect, consistent with findings from another qualitative study, where several patients reported not questioning their diagnosis due to different reasons.^28^ Some had relatives with PALs, others said that the healthcare workers had reinforced their allergy status by accepting it without doing any additional evaluations.^28^ These observations highlight the need to improve healthcare professionals’ awareness of the referral procedure. Educating caregivers to routinely consider re-evaluation and to communicate the option of allergy assessment to patients could ensure that patients receive appropriate treatment. Additionally, distinguishing allergic reactions from non-allergic side effects is essential, as the latter can lead to misinterpretation of reactions.^1,2^ Patients with mild non-allergic side effects can be de-labelled without further allergy work-up, and correctly identifying this patient category is therefore important.^22^

In the present study, participants had experienced misinterpretation by physicians of late-onset reactions, even urticarial reactions occurring several days after therapy start, as unspecific adverse events. This misinterpretation contributed to the re-administration of penicillin and led to a subsequent reaction in some cases. The misinterpretation of mild, non-immediate skin reactions and the lack of PALs in several cases can be attributed to the current Swedish routine in primary care.^29–31^ Current Swedish guidelines advise against labelling for PA if the phenotype is non-specific exanthema without itching; therefore maculopapular rashes are not routinely documented under the allergy labels. In case of mild skin symptoms during a course of penicillin, administration of antihistamines and/or corticosteroids to enable continuation of the treatment, or premedication before a subsequent treatment, are also widely used methods. Referral to an allergy specialist is not advised on a regular basis for mild and late-onset urticaria occurring 72 h or more after the initial dose; instead, a direct challenge should be performed in the primary care setting.^29–31^ However, allergic sensitization to penicillin, even for immediate-type allergy, can occur during the treatment course, and classifying reactions solely based on the time from the first dose may lead to misinterpretation. Once sensitization has occurred, a subsequent dose can trigger an IgE-mediated immediate reaction, including urticaria, angioedema or anaphylaxis. To avoid this limitation, both the timing and the clinical morphology of the reaction should be considered.^32^

All patients who had a planned evaluation were willing to accept a drug challenge test. The participants expressed that they trusted the personnel who performed the evaluations and stated that the competence they witnessed in the allergy clinic played a crucial role in their decision to accept a drug challenge test. Being evaluated as a planned procedure, the potential for minimizing the future risk of MDR bacteria and getting a conclusion were important motivating factors for some of the participants. Our results were in line with a previous study that found that experiencing negative consequences associated with a PA, such as difficulties in planning a surgical procedure, not being able to be treated with first-line antibiotics, and experiencing side effects due to broad-spectrum antibiotics, all had a great impact on patients and motivated them to accept allergy evaluation.^16^ Participants who were aware that penicillin is first-line treatment for most community-acquired infections accepted that having access to penicillin would make the choice of antibiotics easier. The majority of patients did not feel particularly stressed about the drug challenges but underlined that they were only willing to perform it at the allergy clinic and not elsewhere.

The participants expressed willingness to accept penicillin following a negative challenge, although several participants preferred to receive the initial course in a location with advanced healthcare infrastructure due to anxiety related to previous negative experiences. Additionally, the correct documentation after a negative challenge or a confirmed PA was highlighted by several participants as important for feeling safe in taking antibiotics in future.

### Strengths and limitations

Participants in this study were interviewed, which provided more detailed information and deeper understanding of the patients’ perspective compared with a questionnaire study. The respondents belonged to different risk groups regarding their suspected PA and were at different stages of the allergy assessment. These different categories of patient yielded a broader understanding of the patients’ views.

There were also limitations to this study. One could be the sample size. However, other studies have shown that saturation can be achieved in a narrow range of interviews in studies with a relatively homogeneous study population, as in our study.^24^ Although we enrolled both women and men with different phenotypes of reactions, all were native Swedish speakers, and patients who were not Swedish speakers or had Swedish as a second language were not interviewed. Future studies might address the perspective of these latter groups to improve the clinical care of all patients who undergo evaluation for suspected drug allergy. Those patients who participated in this study were evaluated according to a risk stratification tool that is based on updated Danish guidelines.^1^ Prolonged challenges were offered from the second day if drug challenge on the first day was negative according to the Danish guidelines.^22^ As prolonged challenges are not routinely used in Sweden or other countries and there is no consensus on a preferred procedure, our findings regarding willingness to take penicillin after a negative prolonged challenge cannot be applied to those who perform single-dose challenges or titrated challenges without further doses.^32^

European guidelines reviewing 6484 patients found that 2.3% were positive on initial challenge and 5.5% during prolonged challenges, but concluded there is no consensus on a preferred procedure and made no recommendation for or against prolonged oral challenge.^32^ One advantage of prolonged challenges is that non-immediate reactions are often T-cell mediated and may require extended or repeated drug exposure to manifest. Although most reactions are mild maculopapular rashes, some can persist for weeks and require corticosteroid treatment. Prolonged challenge enables identification of these reactions and allows patients to be cautioned against future exposure.^1,33^ A potential disadvantage can be that prolonged challenges contribute to antimicrobial resistance.^1^ Another important aspect to consider is that prolonged challenges may convince the patient to take penicillin again. A single dose may not be sufficient to convince them, and then they may continue to contribute to resistance by avoiding penicillin. Future research should explore de-labelling of penicillin allergies to determine whether prolonged challenges improve sensitivity or not.

In conclusion, our study aimed to contribute to the field by drawing attention to several factors that may support more effective de-labelling. For example, helping patients understand the potential negative consequences of inaccurate allergy labels may influence their willingness to undergo evaluation, and strengthening the referral process may further improve de-labelling outcomes. Our findings also suggest a new insight: patients appreciated being close to well-developed healthcare services during their first penicillin use after de-labelling, indicating that structured primary care follow-up may help facilitate penicillin use.

We hope that these considerations can be integrated into clinical practice to promote safer antibiotic use and more successful de-labelling. Education of non-allergists so that they can become more actively involved in evaluating patients with low-risk reactions is a key factor in increasing de-labelling rates, although high-risk reactions should continue to be assessed by allergy specialists.

## Supplementary Material

dlaf261_Supplementary_Data

## Supplementary data

Table S1 and Supplementary material are available as Supplementary data at *JAC-AMR* Online.
