# Correction to: Patients’ experiences of penicillin allergy evaluation: a qualitative study

**DOI:** 10.1093/jacamr/dlag143

**Published:** 2026-07-17

This is a correction to: Ágnes Csuth, Linda Gustafsson Wännlund, Maria C Jenmalm, Lene Heise Garvey, Charlotte Angelhoff, Patients’ experiences of penicillin allergy evaluation: a qualitative study, *JAC-Antimicrobial Resistance*, Volume 8, Issue 1, February 2026, dlaf261, https://doi.org/10.1093/jacamr/dlaf261

In the originally published version of this manuscript, the institutional affiliations listed for the second and last authors were incorrect.

Their affiliations have been corrected to read:

^2^Clinical Department of Allergy Center in Linköping, Region Östergötland, Linköping, Sweden

^3^Department of Health, Medicine, and Caring Sciences, Linköping University, Linköping, Sweden

Instead of:

^1^Division of Inflammation and Infection, Department of Biomedical and Clinical Sciences, Linköping University, Linköping, Sweden

^3^Department of Health, Medicine, and Caring Sciences, Linköping University, Linköping, Sweden

