# Supplementary material for: Patients’ experiences of penicillin allergy evaluation: a qualitative study
Source: JAC Antimicrob Resist. 2026 Feb 4;8(1):dlaf261. doi: 10.1093/jacamr/dlaf261 (PMC12870118; doi:10.1093/jacamr/dlaf261)
Supplement: dlaf261_Supplementary_Data [file dlaf261_supplementary_data.docx]

**Supplementary material**

**Definitions**

- Immediate reaction: a reaction developing within less than 2 hours after exposure.

- Non-immediate reaction: a reaction developing two hours or later after exposure.

- Systemic reaction: a reaction that includes *e.g.* anaphylaxis, urticaria, angioedema - i.e. involvement of more than just a local site.

**Evaluation of patients according to the study protocol**

The chronology, phenotype, treatment of the reaction, duration, and comorbidities were discussed at the first visit, and the patients were categorized into different risk groups according to the risk stratification table based on the updated Danish guidelines (Supplementary Table 1).^1^ Those with a high risk for anaphylaxis due to immediate-type reactions or severe underlying comorbidities were evaluated with specific IgEs towards penicillins (penicilloyl-V and -G, amoxicillin, ampicillin, and a mix of minor determinants). If any specific IgE test was positive (>0.35 kU/L), the patient from this risk group was considered penicillin allergic.

If the specific IgE assays were negative, titrated skin tests - including skin prick tests and titrated intradermal tests - were carried out with the culprit drug, and in cases of negative laboratory and skin tests, a titrated provocation towards the culprit was performed.

Patients with moderate maculopapular exanthema who had been treated with oral steroids, and/or whose symptoms lasted at least 7 days, were evaluated with non-titrated intradermal tests towards the culprit drug and a panel of other penicillins consisting of benzylpenicillin, amoxicillin, ampicillin, and cloxacillin. If results were negative, a non-titrated drug challenge towards the culprit was performed. All intradermal tests included an immediate reading after 20 minutes and a delayed reading for 7 days.

Patients with a low risk for anaphylaxis underwent a challenge with a therapeutic dose of the culprit penicillin without prior laboratory testing or skin testing.

Patients who experienced non-allergic side effects without skin or mucosal involvement, or who had already tolerated the suspected medication, underwent a non-titrated challenge towards the culprit.

All patients were evaluated towards the culprit penicillin, or if this was unknown, the work-up was performed with amoxicillin.

If the drug challenge was negative on the first day, a prolonged provocation was carried out from the second day for 4 days in all risk groups, except those in whom penicillin allergy was considered unlikely. All patients who underwent drug challenge were advised to take pictures of skin symptoms, finish the prolonged provocation and contact the clinic in case of a reaction, and a telephone appointment was routinely scheduled 4 weeks after the challenge for those who did not contact the clinic. At these appointments, the outcome was discussed, further investigations were offered when needed, the allergy label was removed or updated according to the results, and a report was sent to the referring physician.

In some cases—when skin testing or drug challenge was positive for a penicillin with an extended spectrum—further evaluation for narrow spectrum penicillins was offered; however, evaluation of cross-reactivity was not part of this study.

The patients who participated in this qualitative study were assessed due to reactions to phenoxymethylpenicillin, amoxicillin, flucloxacillin, and unspecified penicillin.

| **High risk for an allergic reaction** | **Low risk for anaphylaxis**  *Exclude that the patient has had life-threatening symptoms.*  *In cases of severe comorbidity (severe cardiovascular or respiratory disease, systemic mastocytosis), manage the patient as high-risk.* | **Penicillin allergy is unlikely**  *Exclude that the patient has had life-threatening symptoms or involvement of the skin and mucous membranes.* |
| --- | --- | --- |
| High risk for anaphylaxis:  (At least one criterion is met)   - Symptom onset within 2 hours after the first dose - Bronchospasm/hoarseness/stridor - Syncope/hypotension - Treatment with adrenaline or suspected anaphylaxis - Need for hospital care during an acute reaction - Urticaria/angioedema/intense itching   Moderate non-immediate reaction:   - Maculopapular rash lasting at least 7 days or requiring oral corticosteroids (delayed reaction) | - Onset of symptoms >2 h after first dose   **And**   - Benign skin rashes, such as exanthema, flushing, mild itching, and/or maculopapular rashes lasting <7 days and did not require oral corticosteroids.   **or**   - Unclear medical history regarding the chronology, symptoms, and treatment of the index reaction. It can be ruled out that the reaction was severe (no need for ambulance transport, hospitalization, adrenaline, corticosteroids, etc.). | - Mild non-allergic symptoms (e.g., isolated gastrointestinal symptoms, headache, fungal infection, fatigue, etc.) - Penicillin hypersensitivity only in a family member, not in the patient - The exact same medication has been taken after the reaction without any reaction - Symptom onset before the start of antibiotic treatment |
| Referral to dermatologist:   - Severe skin symptoms (blistering, widespread scaling, mucous membrane involvement) - Suspected serum sickness/joint involvement - General malaise with fever - Vasculitis - Suspected DRESS/AGEP/SJS/TEN - Eosinophilia |  |  |
| No allergy work-up:   - Potentially life-threatening organ involvement, such as bone marrow, liver, kidney, lung, or heart involvement, or other severe hematological abnormalities - Positive specific IgE (against penicillins)⃰ or strong anamnestic certainty - The patient declines further investigation |  |  |

**Supplementary Table 1. Risk stratification table** (modified from Tannert LK *et al*.)

⃰Testing for specific IgEs for penicillin is only recommended for patients with immediate-type reactions or with severe co-morbidities.

**References for the supplementary material**

1. Tannert LK, Mosbech H, Hjortlund J *et al.* [Work-up of penicillin allergy]. Ugeskr Laeger. 2020; **182**: V02200116
